# Sample size in multistakeholder Delphi surveys: at what minimum sample size do replicability of results stabilize?

**DOI:** 10.1016/j.jclinepi.2024.111485

**Published:** 2024-07-26

**Authors:** Anthony Muchai Manyara, Anthony Purvis, Oriana Ciani, Gary S. Collins, Rod S. Taylor

**Affiliations:** a School of Health and Wellbeing, University of Glasgow, Glasgow, UK; b Global Health and Ageing Research Unit, Bristol Medical School, University of Bristol, Bristol, UK; c Centre for Research on Health and Social Care Management, SDA Bocconi School of Management, Milan, Italy; d UK EQUATOR Centre, Centre for Statistics in Medicine, Nuffield Department of Orthopaedics, Rheumatology & Musculoskeletal Sciences, University of Oxford, Oxford, UK; e https://ror.org/https://ror.org/02v3sdn51 MRC/CSO Social and Public Health Sciences Unit & Robertson Centre for Biostatistics, School of Health and Wellbeing, University of Glasgow, Glasgow, UK

**Keywords:** Delphi, Sample size, Replicability, Resampling, Stability, Consensus, Expert panel, Quantitative research, Qualitative research

## Abstract

**Background and Objective:**

The minimum sample size for multistakeholder Delphi surveys remains understudied. Drawing from three large international multistakeholder Delphi surveys, this study aimed to: 1) investigate the effect of increasing sample size on replicability of results; 2) assess whether the level of replicability of results differed with participant characteristics: for example, gender, age, and profession.

**Methods:**

We used data from Delphi surveys to develop guidance for improved reporting of health-care intervention trials: SPIRIT (Standard Protocol Items: Recommendations for Interventional Trials) and CONSORT (Consolidated Standards of Reporting Trials) extension for surrogate end points (*n* = 175, 22 items rated); CONSORT-SPI [CONSORT extension for Social and Psychological Interventions] (*n =* 333, 77 items rated); and core outcome set for burn care (*n* = 553, 88 items rated). Resampling with replacement was used to draw random subsamples from the participant data set in each of the three surveys. For each subsample, the median value of all rated survey items was calculated and compared to the medians from the full participant data set. The median number (and interquartile range) of medians replicated was used to calculate the percentage replicability (and variability). High replicability was defined as ≥80% and moderate as 60% and <80%

**Results:**

The average median replicability (variability) as a percentage of total number of items rated from the three datasets was 81% (10%) at a sample size of 60. In one of the datasets (CONSORT-SPI), a ≥80% replicability was reached at a sample size of 80. On average, increasing the sample size from 80 to 160 increased the replicability of results by a further 3% and reduced variability by 1%. For subgroup analysis based on participant characteristics (eg, gender, age, professional role), using resampled samples of 20 to 100 showed that a sample size of 20 to 30 resulted to moderate replicability levels of 64% to 77%.

**Conclusion:**

We found that a minimum sample size of 60–80 participants in multistakeholder Delphi surveys provides a high level of replicability (≥80%) in the results. For Delphi studies limited to individual stakeholder groups (such as researchers, clinicians, patients), a sample size of 20 to 30 per group may be sufficient.

## 1 Background

The Delphi methodology uses Wisdom of Crowds theory, that is, collective intelligence of a group of people is superior to individual wisdom [1,2]. Introduced in the 1950s for forecasting issues of interest to the US military, the Delphi methodology has since evolved and has been used in many fields including health research [3]. The methodology involves use of experts as participants; maintaining anonymity between participants; iterations (>1 survey round) and controlled feedback (to allow for ‘communication’ between participants); and aggregating participants’ collective opinion [4–7]. It has been used to build consensus in various exercises such as needs assessment, policy determination, estimation of disease prevalence, and development of clinical and health reporting guidelines [6–9]. Despite diverse use, there remains a lack of standardization in Delphi methodology [10,11]. One of these methodological areas is the minimum sample size for such studies. Given the qualitative nature of the Delphi methodology (aggregation and convergence of opinion), formal sample size calculation is often perceived as not necessary: however, the number and characteristics of participants need to be carefully considered [4,6,7,12]. For specific topics or research areas, multistakeholder Delphi surveys (ie, recruiting various stakeholders) should be recruited for representativeness, for example, the appropriate range of stakeholder and user groups and geographies [8,13].

A recent review of systematic reviews of Delphi methodology found the sample size in most Delphi studies was small (<20 participants) to medium (≤40 participants) [11]. Such small numbers call into question the application of Wisdom of Crowds theory, validity, and reliability of findings [11]. On the other hand, large sample sizes may have diminishing returns in studies that are qualitative in nature, particularly after data saturation point is reached, that is, point where addition of more participants does not generate new insights [14]. However, large sample sizes could benefit from wider engagement of stakeholders leading to endorsement of Delphi survey outputs. Although a minimum sample size of 10 to 25 participants has been suggested [15–17], there is a lack of empirical research to justify this. Furthermore, sample sizes of Delphi surveys likely depend on the specific topic under investigation and heterogeneity of participants [4,12]. In 2005, Akins et al applied a bootstrapping application and found that results stability could be achieved with as few as 23 participants [16]. However, this bootstrapping analysis was drawn from a small sample of 23 participants with similar training and understanding of the field under study [16].

In 2016, Yoshida et al used bootstrapping in the Child Health and Nutrition Research Initiative (CHNRI) methodology (that applies Wisdom of Crowds theory and used to rate research priorities), to understand collective opinion characteristics in a sample of 90 participants. They found that stability of responses (ie, a point where adding more participants did not significantly change ranked priorities) was achieved with a sample of 45 to 55 experts [14]. However, while the CHNRI is a methodology of summarizing collective opinion, it remains unknown if findings on minimum sample size can be extrapolated to Delphi surveys.

This study aimed to use Delphi surveys recruiting international and multidisciplinary participants to 1) investigate the effect of increasing sample size on replicability and stability of results; and 2) assess whether the level of replicability and stability of results differed with participant characteristics: gender, profession, years of experience, and geographical location.

## 2 Methods

### 2.1 Delphi surveys used

Data from the first round of three large and multistake-holder Delphi surveys carried out in the development of trial reporting guidelines and a core outcome set were used. The reporting guidelines were extensions of the Consolidated Standards of Reporting Trials (CONSORT) and SPIRIT (Standard Protocol Items: Recommendations for Interventional Trials) checklists for reporting of randomized trials: SPIRIT-Surrogate and CONSORT-Surrogate extensions for trials using surrogate end points as primary outcomes [18–20]; and CONSORT-SPI (CONSORT extension for trials of Social and Psychological Interventions) [21,22]. The core outcome set was the Core Outcome Set in Burn Care Research international (COSB-i) [23]. Characteristics of these Delphi surveys are briefly described.

In the SPIRIT|CONSORT-Surrogate Delphi survey, 212 eligible participants registered to participate in the survey, of which 195 (92%) provided ratings in the first round. Participation was drawn from 30 countries; multidisciplinary, with representation from over 26 disease and research areas; and represented a diverse group of stakeholders, including trial investigators, methodologists and managers, clinicians and allied health professionals, statisticians, surrogate content experts, journal editors, patient and public partners, regulators, health technology assessment experts, ethics committees, and funding panel members. Participants rated 22 items (related to both SPIRIT and CONSORT checklists) between August and October 2022 on a 9-point Likert scale.

The CONSORT-SPI Delphi survey invited ~1500 participants, 584 responding to the invitation, of which 384 (66%) eligible participants completed the first round. Participation was drawn from 32 countries and multidisciplinary including researchers, funders, policy makers, SPI’ practitioners, providers, and end-users. Participants rated 77 items for CONSORT extension checklist using a 10-point Likert scale between September and October 2013.

In the COSB-i Delphi survey, 794 participants from 77 countries comprising of clinicians, researchers, patients, and carers took part in round one. Participants rated 88 items on a 9-point Likert scale between October 2018 and July 2019.

### 2.2 Statistical analyses

We excluded from the final analysis participants who answered ‘unsure/no opinion’ or did not rate all items. The sample sizes used were 175 for SPIRIT|CONSORT-Surrogate, 333 for CONSORT-SPI and 553 for COSB-i. All analyses were carried out on the R programming language version 4.2.0 (https://cran.r-project.org/).

#### 2.2.1 Replicability and stability definition and presentation

Resampling with replacement was used to draw random subsamples from the pool of participants. For each resampled sample, the median value of a Likert scale for each survey item was calculated and compared to the median of each corresponding survey item using the full pool of participants. The number of medians from the resampled sample that were the same as from the full sample were then determined. The median (and interquartile range [IQR]) and mean of these medians was then computed. In total, 1000 samples were resampled from the original data for each sample size ranging from 20 to 500. This process was then repeated for the means of each rated survey item in the three datasets. Replicability is presented in medians and IQR (ie, number of medians replicated for each resampled sample when compared to the full sample), percentage median replicability (medians replicated/number of items rated in the survey) and percentage variability (IQR /number of items rated in the survey). We defined high replicability as the sample size resulting to 80% replicability of the medians rated in the full sample based on the average of the three datasets and sample size when all three datasets had an 80% replicability. This allowed us to give a range rather than a specific sample size that would result to high replicability consistent with lack of formal sample size calculation in studies that are qualitative in nature. Further, replicability between 60% and <80% was defined as moderate while ≥90% was defined as very high.

#### 2.2.2 Subgroup analyses

Subgroup analyses were carried out depending on the data available in each of the survey datasets. In the SPIRIT CONSORT-Surrogate dataset, participants originally classified themselves into professional roles: Epidemiologist, Trial Investigator, Statistician, Trial Methodologist or Clinician/Health and allied health professional. To categorize these roles more efficiently, we aligned Epidemiologists and Trial Investigators as “Researchers”, Statisticians and Trial Methodologists as “Methodologists” and Clinician/Health and allied health professionals as “Clinicians”. In addition, participants were also split into two groups based on the number of years’ experience they have in their current role: ‘less than 15 years’ experience’ and ’ 15 or more years’ experience’. Similarly, in the CONSORT-SPI dataset participants were grouped by age: ‘44 years old or younger’ and ‘45 years old and above’, gender: ‘men’ or ‘women’ and by country. Participants came from 32 countries so to balance this demographic evenly; participants were allocated into one of two groups: ‘Europe’ or ‘Rest of the World’. This was repeated in the COSB-i dataset for participants’ locations. Participants were also grouped by participant type: ‘patient and carer’ or ‘clinician/health care professional’ and by country income as classified by the World Bank Group: ‘High Income Country’, ‘High Middle-Income Country’, ‘Low Middle-Income Country’ and ‘Low Income Country’.

#### 2.2.3 Consensus replicability and consensus

As a consensus building exercise, Delphi surveys often define consensus criteria a priori. For example, recent health research reporting guidelines have defined consensus as: consensus for inclusion: ≥70% participants scoring an item as critical (eg, 7-9 on a 9-point Likert scale) and <15% scoring an item as not important (eg, 1-3 on a 9-point Likert scale); and consensus for exclusion: ≥70% scoring an item as not important and <15% scoring an item as critical [24–27]. We, therefore, did an additional analysis to determine change in number of items reaching consensus with increasing sample size. Similar to the replication of medians, we calculated the number of items reaching consensus in each of the Delphi surveys. Using 1000 random samples ranging from 20 to 500 from each Delphi survey, we determined the median number of items reaching consensus at each sample size and presented using boxplots.

## 3 Results

### 3.1 Replicability in full sample

Figure shows replicability of results with increasing sample size in the three datasets: random resampled samples (20-500) on the x-axis and the median number of medians replicated, median replicability, on the y-axis. Generally, the median replicability increased, and variability (IQR) decreased with increasing sample size; however, replicability stabilized in certain sample sizes, that is, increase in sample size did not improve replication of results (Fig). Table 1 shows the percentage median replicability and percentage variability. On average, at a sample size of 20, the replicability was 68% (with a variability of 12%). The average replicability increased to 76% (10%) in a sample size of 40 and reached a high replicability level (≥80%) at a sample size of 60; although in one of the datasets (CONSORT-SPI), the high replicability level was reached with a sample size of 80. Replicability of results stabilized after a sample size of 80. On average, increasing the sample size from 80 to 160 increased the replicability by only 3% and reduced variability by 1%. Supplementary Table A.1 shows the median and percentage replicability in the three datasets from sample size 20 to 500 with step increases of 10. Supplementary Figure A.1 shows mean replicability of the medians of rated items with increasing sample size in the three datasets.

### 3.2 Replicability in stakeholder groups

Tables 2-5 show results of subgroup analyses comparing replicability levels based on various participant characteristics. Replicability levels were similar between men and women: 70% at a sample size of 20 and reaching 80% at a sample size of 50, see Table 2.

Table 3 shows the replicability levels with increasing sample sizes in researchers, methodologists, and clinicians in the SPIRIT|CONSORT-Surrogate dataset. At any sample size, researchers had a ≥4% lower replicability compared to methodologists or clinicians. At a sample size of 20, there was a 64% replicability level among researchers compared to 68% among methodologists and clinicians. Increasing the sample size to 80 resulted to a replicability of 73% among researchers and 86% among methodologists and clinicians.

Table 4 shows replicability in patients/carers and clinicians/health professionals based on the COSB-i dataset. The replicability levels were generally similar between the two groups: 68% in patients/carers at a sample size of 20% and 66% in clinicians/health professionals which increased to 80% and 81%, respectively, at a sample size of 60.

Table 5 shows replicability levels with increasing sample sizes in participants with <15 years of experience compared to participants with ≥15 years of experience in the SPIRIT|CONSORT-Surrogate dataset and participants aged less than 44 years with those aged 44 years or more in the CONSORT-SPI dataset. At any given sample size, replicability was ≥4% higher in participants with <15 years of experience compared to those with ≥15 years of experience. Replicability stabilized at a sample size of 60 in both groups. However, differences observed with experience level were not replicated when age was used as a proxy for years of experience in the CONSORT-SPI dataset. Replicability was generally similar in all sample sizes; starting with 70% at a sample size of 20 in participants of ≤44 years of age and 69% in participants of >44 years of age and reaching 81% in both groups at a sample size of 50 (Table 6).

Supplementary Table A.2 comparing replicability levels with increasing sample size in participants from the four types of income level countries in the COSB-i dataset.

### 3.3 Replicability in achievement of consensus

Supplementary Figure A.2 shows the median number of items reaching consensus with increasing sample size and Table 6 shows the percentage median (and percentage variability) of items reaching consensus in all rated items with increasing sample size in the three datasets. While the median number of items reaching consensus was relatively similar across the subsamples, the variability decreased with increasing sample size.

## 4 Discussion

Using three large, and multidisciplinary Delphi surveys involving a total of > 1000 participants, we quantified the effect of increasing sample size on replicability of results and whether replicability differed with participant characteristics. Drawing resampled samples of 20-500, we found that a high replicability level (≥80%) on medians of rated items was reached at a sample size of 60 to 80 participants. Further increase in sample size resulted to modest increase in replicability levels reaching 90% at a sample size of 200. Samples of 20 to 40 participants resulted to moderate replicability levels of 67%-76%. Additionally, replicability stabilized at specific sample sizes. Subgroup analyses based on participant characteristics using resampled samples of 20 to 100 found that a sample of 20 to 30 resulted to moderate replicability levels of 64% to 77%. Our findings on replicability levels and stability were in part consistent with bootstrapping application on the CHNRI methodology which uses Wisdom of Crowds to rank research priorities. Sample size to reach a replicability of 80% in rated items was an average of 60-80 participants in our study and 85 participants in the CHNRI methodology study [14]. Replicability increased with sample size and stabilized in specific sample sizes in both studies; however, these specific sample sizes differed slightly in the two studies.

While a sample size of 60-80 participants would on average result to a high replicability (of 80%) of the results from an otherwise larger sample, increasing the sample size above this may have benefits. In this study, we found that increases in sample size resulted to higher replicability levels in all three Delphi surveys, albeit with points of stability in replicability, suggesting improved reliability and validity. Furthermore, it can be argued that larger sample sizes result to ownership of Delphi output by more people and consequently helping with output implementation compared to modest samples. For example, participants taking part in a Delphi survey to develop a reporting guideline may identify with the guideline and hence use it themselves or recommend it to others. However, evidence to support this argument is needed; and there may be other ways to increase implementation of research outputs. Additionally, apart from rating items, Delphi surveys have been used to solicit additional important items not contained in the survey [18], hence a larger sample provides a bigger pool of experts to solicit for new items. Finally, our findings imply that in instances where consensus is more difficult, sample sizes need to be larger. On the flip side, while large Delphi samples can have benefits, researchers should be aware of diminishing returns with increasing sample size that is, recruiting more participants may not alter the aggregate item level Delphi ratings. Furthermore, participant recruitment and retention in Delphi surveys can be challenging and time consuming, and participants are often experts with competing tasks for their time. Therefore, some Delphi surveys may only be able to use small sample sizes. Consistent with studies on optimal sample sizes in qualitative or opinion aggregation studies [14,28], this study confirms that even small sample sizes can provide substantially valid and reliable findings. Furthermore, the reliability, validity and consensus from small Delphi samples can be improved through subsequent consensus building exercises, such as discussion and voting in consensus meetings. However, from our experience, gaining consensus on most items rated in a Delphi survey, which can be facilitated by a large sample, allows for having a shorter agenda for consensus meetings resulting to more efficiency and productivity from such meetings whose planning and conducting is resource intensive.

Another important consideration for multistakeholder Delphi surveys is the sample size of stakeholder groups included. From our analyses, we propose that a sample size of 20-30 for each stakeholder group may be sufficient. We found that such a sample size (based on any participant characteristic explored) resulted to moderate replicability levels (64%−77%). Such a sample size may be sufficient given that agreement across stakeholder groups may be considerably similar in most rated items. For example, a secondary analysis of one of the datasets used in this study (COSB-i) found that there was considerable agreement between participants from low and middle income countries and high income countries: > 90% agreement on all items rated [29].

The approach used in our study could be use in Delphi to monitor stability of results over time and inform halting or diversification of the sample. However, as highlighted earlier, there are other benefits to recruiting a larger sample beyond stabilization of results. This study findings can be used to inform Delphi target sample size using the estimates of replicability levels associated with each sample size. However, use of these estimates should be done along with other considerations. First, response rates and sample size attrition across Delphi rounds should be considered when determining the target sample size. Recent Delphi surveys to develop reporting guidelines have had a response rate of 61%-93% of the invited participants and an attrition (in the context of completing all Delphi rounds) of 11%-33% [25,30–32]. Second, diversity of participants in multistakeholder and international Delphi surveys is vital. Therefore, researchers may find it useful to diversify their sample rather than just increasing numbers of participants from one stakeholder group or geographical region. Furthermore, the validity and reliability of Delphi findings will depend on the expertise of included participants [4–6,12] including patient and public partners, research output end users, and consumers [11,33,34]. Therefore, researchers should define and adhere to an inclusion criteria list during recruitment to ensure those participating contribute expertise to the Delphi process. Finally, success of Delphi surveys can be argued to mainly be about implementation of survey output and subsequent impact on outcomes rather than conduct of the survey [10] including use of large sample sizes. Researchers should, therefore, balance time and resource investment between participant mobilization and recruitment and later dissemination and implementation of the survey output.

This study uses three large multistakeholder and international Delphi surveys using 9 and 10-point Likert scales to inform replicability and stability with increasing samples. Another strength of our study is that Delphi surveys used had varied number of items for rating (22 to 88 items) and results remain very similar. However, it remains unknown whether our findings are generalizable to Delphi studies using lower point Likert scales, different number of items to rate or different topics to address. However, we used Delphi studies that looked at three different topics (surrogate outcomes, SPI. and burn care) and rated different number if items ranging from 22 to 88. Finally, the study was not reported using any relevant reporting guidelines (as we are not aware of any) neither the protocol preregistered on any platform.

## 5 Conclusions

In conclusion, a sample size of 60 to 80 participants rating all items in multistakeholder Delphi surveys was shown to result in high levels (≥80%) of replicability. For individual stakeholder subgroups (eg, such as researcher, clinicians, patients), a sample size of 20 to 30 rating all items per group would be enough to provide a moderate replicability as interstakeholder discordance is likely to be low for most rated items in a Delphi survey. Increase in sample size improved the replicability albeit with points of stability in various sample sizes. Furthermore, even modest sample sizes resulted to moderate replicability levels. Our replicability levels with increased sample size provide a resource to inform minimum sample size in future multistakeholder Delphi surveys. However, the final determination of the target sample size needs to also take into account the response rates and attrition between survey rounds; diminishing returns of increasing sample size; and the diversity and expertise of participants.

## Supplementary Material

Supplementary data Supplementary data to this article can be found online at https://doi.org/10.1016/j.jclinepi.2024.111485.

## Figures and Tables

**Figure:**
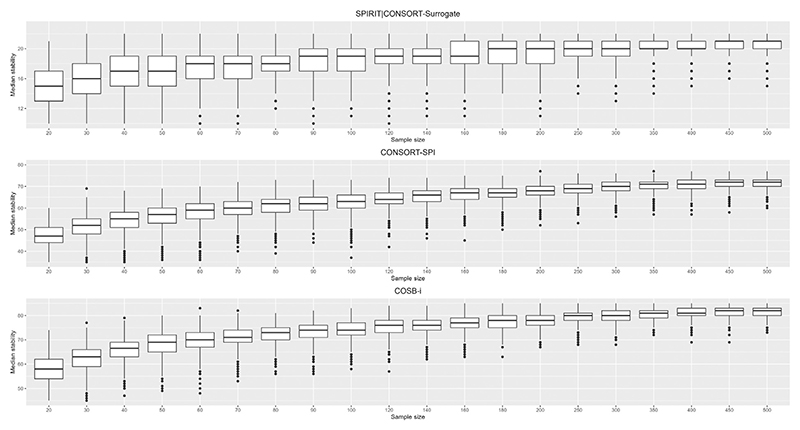
Boxplots showing replicability of results with increasing sample size in the three datasets: random resampled samples (20-500) on the x-axis and the median number of medians replicated (median replicability) from the full sample ratings (y-axis). Note for each panel, the maximum value of the y-axis is the number of items in each survey (22 for SPIRIT|CONSORT-Surrogate, 77 for CONSORT-SPI and 88 for COSB-i, respectively)).

**Table 1:** The percentage median replicability and percentage variability in all rated items with increasing sample size in the three datasets

	SPIRITICONSORT-surrogate *N* = 175		CONSORT-SPI *N* = 333		COSB-i *N* = 553		Average from 3 datasets
	Items rated = 22		Items rated = 77		Items rated = 88		
Sample size	% median replicability (variability)		% median replicability (variability)		% median replicability (variability)		% average median replicability (variability)
20	68% (18%)		61% (9%)		73% (9%)		67% (12%)
30	73% (18%)		68% (9%)		78% (8%)		73% (12%)
40	77% (14%)		71% (9%)		81% (7%)		76% (10%)
50	77% (18%)		74% (9%)		83% (5%)		78% (11%)
60	82% (14%)		77% (10%)		84% (5%)		81% (10%)
70	82% (14%)		78% (8%)		85% (6%)		82% (9%)
80	86% (9%)		81% (8%)		86% (6%)		84% (8%)
90	86% (14%)		81% (8%)		86% (5%)		84% (9%)
100	86% (14%)		82% (8%)		88% (5%)		85% (9%)
120	86% (9%)		83% (6%)		89% (6%)		86% (7%)
140	86% (14%)		86% (6%)		90% (5%)		87% (8%)
160	86% (9%)		86% (7%)		90% (4%)		87% (7%)
180	91% (13%)		87% (6%)		90% (4%)		89% (8%)
200	91% (9%)		88% (7%)		91% (4%)		90% (7%)
250	91% (9%)		90% (5%)		92% (3%)		91% (6%)
300	91% (9%)		91% (4%)		92% (4%)		91% (6%)
350	91% (9%)		92% (4%)		93% (3%)		92% (5%)
400	91% (4%)		92% (5%)		93% (2%)		92% (4%)
450	95% (4%)		94% (4%)		93% (3%)		94% (4%)
500	95% (4%)		94% (5%)		93% (3%)		94% (4%)

**Table 2:** The median (interquartile range) replicability and percentage replicability (variability) in all rated items in men and women in the CONSORT-SPI dataset

Sample size	CONSORT-SPI				
	Men, *N* = 171			Women, *N* = 162	
	Median replicability (IQR)	% replicability (%variability)		Median replicability (IQR)	% replicability (%variability)
20	54 (8)	70% (10%)		54 (7)	70% (9%)
30	58 (7)	75% (9%)		58 (7)	75% (10%)
40	60 (7)	78% (9%)		61 (6)	79% (8%)
50	62 (6)	81% (7%)		63 (5)	82% (6%)
60	64 (6)	83% (8%)		64 (5)	83% (7%)
70	65 (6)	84% (7%)		65 (4)	84% (5%)
80	66 (5)	86% (6%)		66 (4)	86% (5%)
90	67 (5)	87% (7%)		67 (4)	87% (6%)
100	67 (5)	87% (7%)		67 (4)	87% (6%)

IQR, interquartile range.

**Table 3:** The median (interquartile range) replicability and percentage replicability (variability) in all rated items among researchers, methodologists, and clinicians in the SPIRIT|COSORT-SURROGATE dataset

Sample size	SPIRITICONSORT-surrogate							
	Researchers, *N* = 34			Methodologists, *N* = 53			Clinicians, *N* = 29	
	Median replicability (IQR)	% replicability (variability)		Median replicability (IQR)	% replicability (variability)		Median replicability (IQR)	% replicability (variability)
20	14 (4)	64% (18%)		15 (3)	68% (14%)		15 (4)	68% (18%)
30	15 (3)	68% (14%)		17 (3)	77% (14%)		16 (4)	73% (18%)
40	15 (3)	68% (14%)		18 (3)	82% (14%)		17 (4)	77% (18%)
50	15 (3)	68% (14%)		18 (3)	82% (14%)		18 (3)	82% (14%)
60	16 (3)	73% (14%)		18 (2)	82% (9%)		18 (3)	82% (14%)
70	16 (3)	73% (14%)		19 (3)	86% (14%)		20 (3)	82% (14%)
80	16 (3)	73% (14%)		19 (2)	86% (9%)		20 (3)	86% (14%)
90	16 (3)	73% (14%)		19 (2)	86% (9%)		20 (2)	86% (9%)
100	17 (3)	77% (14%)		19 (2)	86% (9%)		20 (3)	86% (14%)

IQR, interquartile range.

**Table 4:** The median (interquartile range) replicability and percentage replicability (variability) in all rated items among patients or careers and clinicians or health professionals in the COSB-i dataset

Sample size	COSB-i				
	Patients/Carers, *N* = 72			Clinicians/Health professionals, *N* = 481	
	Median replicability (IQR)	% Replicability (variability)		Median replicability (IQR)	% Replicability (variability)
20	60 (9)	68% (10%)		58 (8)	66% (9%)
30	64 (9)	73% (10%)		64 (7)	73% (8%)
40	67 (7)	76% (8%)		67 (7)	76% (8%)
50	69 (7)	78% (8%)		69 (6)	78% (7%)
60	70 (6)	80% (7%)		71 (5)	81% (6%)
70	72 (5)	82% (6%)		72 (5)	82% (6%)
80	72 (6)	82% (7%)		73 (4)	83% (5%)
90	74 (5)	84% (5%)		74 (5)	84% (5%)
100	74 (6)	84% (7%)		75 (5)	85% (6%)

IQR, interquartile range.

**Table 5:** The median (interquartile range) replicability and percentage replicability (variability) in all rated items comparing participants with less than 15 years of experience with those with 15 or more years of experience in the SPIRIT|CONSORT-Surrogate dataset and participants aged less than 44 years with those aged 44 years or more in the CONSORT-SPI dataset

Sample size	SPIRITICONSORT-surrogate						CONSORT-SPI				
	<15 years experience, *N* = 61			≥15 years experience, *N* = 84			≤44 years old, *N* = 139			>44 years old, *N* = 194	
	Median replicability (IQR)	% Replicability (%variability)		Median replicability (IQR)	% Replicability (%variability)		Median replicability (IQR)	% Replicability (%variability)		Median replicability (IQR)	% Replicability (%variability)
20	15 (3)	68% (14%)		14 (3)	64% (14%)		54 (7)	70% (9%)		53 (9)	69% (12%)
30	16 (3)	73% (13%)		15 (3)	68% (13%)		58 (6)	75% (8%)		57 (7)	74% (9%)
40	17 (3)	77% (14%)		16 (4)	73% (18%)		60 (6)	78% (8%)		60 (6.25)	78% (8%)
50	17 (2)	77% (9%)		16 (3)	73% (14%)		62 (5)	81% (6%)		62 (6)	81% (8%)
60	18 (3)	82% (13%)		17 (3)	77% (14%)		63 (4)	82% (5%)		63 (6)	82% (8%)
70	18 (3)	82% (13%)		17 (3)	77% (14) %		64 (5)	83% (7%)		64 (4.25)	83% (5%)
80	18 (2)	82% (9%)		17 (2)	77% (9%)		65 (5)	84% (6%)		65 (5)	84% (6%)
90	18 (2)	82% (9%)		17 (3)	77% (13%)		65 (4)	84% (5%)		66 (5)	86% (6%)
100	18 (2)	82% (9%)		17 (3)	77% (13%)		66 (5)	86% (6%)		67 (4)	87% (5%)

IQR, interquartile range.

**Table 6:** The percentage median (and percentage variability) of items reaching consensus in all rated items with increasing sample size in the three datasets

Sample size	SPIRITICONSORT-surrogate *N* = 175		CONSORT-SPI *N* = 333		COSB-i *N* = 553	Average of the three datasets
	Items rated = 22		Items rated = 77		Items rated = 88	
	% median number of items reaching consensus (variability)		% median number of items reaching consensus (variability)		% median number of items reaching consensus (variability)	
20	64% (15%)		73% (16%)		71% (15%)	69% (15%)
30	64% (14%)		73% (14%)		69% (14%)	69% (14%)
40	64% (14%)		73% (12%)		69% (11%)	69% (12%)
50	64% (9%)		71% (10%)		68% (10%)	68% (10%
60	64% (14%)		71% (10%)		68% (9%)	68% (11%)
70	64% (9%)		73% (9%)		68% (9%)	68% (9%)
80	64% (14%)		71% (9%)		68% (9%)	68% (11%)
90	64% (9%)		71% (8%)		68% (8%)	68% (8%)
100	64% (9%)		71% (8%)		68% (9%)	68% (9%)
120	64% (5%)		71% (8%)		68% (7%)	68% (7%)
140	64% (5%)		71% (8%)		68% (8%)	68% (7%)
160	64% (5%)		71% (8%)		68% (7%)	68% (7%)
180	64% (5%)		71% (8%)		68% (6%)	68% (6%)
250	64% (5%)		71% (5%)		67% (6%)	67% (5%)
300	64% (5%)		73% (4%)		67% (5%)	68% (5%)
350	64% (5%)		73% (4%)		67% (5%)	68% (5%)
450	64% (5%)		71% (4%)		67% (5%)	67% (5%)
500	64% (5%)		73% (4%)		67% (5%)	68% (5%)

## Data Availability

The SPIRIT|CONSORT-Surrogate dataset will be archived in a repository after publication of key articles. The CONSORT-SPI dataset is accessible via the UK Data Service, https://reshare.ukdataservice.ac.uk/851981/ and the COSB-i dataset via the Dryad platform, https://datadryad.org/stash/dataset/doi:10.5061/dryad.79cnp5htr.

## What is new?

### Key findings

- Compared to actual sample size recruited, the average median replicability (variability) of rated items from the three datasets was 81% (10%) at a sample size of 60.
- In one of the datasets (CONSORT-SPI), a ≥80% replicability was reached at a sample size of 80.
- On average, increasing the sample size from 80 to 160 increased the replicability of results by 3% and reduced variability by 1%.
- For subgroup analysis based on participant characteristics (eg, gender, age, professional role) using resampled samples of 20 to 100 showed that a sample of 20−30 resulted to replicability levels of 64% to 77%.

### What this adds to what is known?

- Delphi surveys used the Wisdom of Crowds theory, that is, collective intelligence of a group of people is superior to individual wisdom.
- Formal sample size calculation for Delphi surveys is not perceived necessary; however, the number and characteristics of participants need to be carefully considered.
- Small Delphi sample size numbers may call into question application of Wisdom of Crowds theory, validity, and reliability of findings.

### What is the implication and what should change now?

- Multistakeholder Delphi surveys should aim to recruit a minimum of 60−80 participants and 20−30 for individual stakeholder groups.
