# Supplementary material for: Sample size in multistakeholder Delphi surveys: at what minimum sample size do replicability of results stabilize?
Source: J Clin Epidemiol. Author manuscript; Available in PMC 2025 Jul 21. (PMC7617918; doi:10.1016/j.jclinepi.2024.111485)

**Supplementary File**

**Table A.1: The median (interquartile range) replicated and percentage median replicability (and percentage variability) in all rated items with increasing sample size in the three datasets**

| Sample size | **Delphi surveys** | | | | | | Average % replicability  (% average variability) |
| --- | --- | --- | --- | --- | --- | --- | --- |
|  | **SPIRIT\|CONSORT-Surrogate, items rated = 22** | | **CONSORT-SPI, items rated = 77** | | **COSB-I, items rated = 88** | |  |
|  | Median replicability (IQR) | % Replicability (%variability) | Median replicability (IQR) | % Replicability (%variability) | Median replicability (IQR) | % Replicability (%variability) |  |
| 20 | 15 (4) | 68 (18) | 47 (7) | 61 (9) | 64 (8) | 73 (9) | 67 (12) |
| 30 | 16 (4) | 73 (18) | 52 (7) | 68 (9) | 69 (7) | 78 (8) | 73 (12) |
| 40 | 17 (3) | 77 (14) | 55 (7) | 71 (9) | 71 (6) | 81 (7) | 76 (10) |
| 50 | 17 (4) | 77 (18) | 57 (7) | 74 (9) | 73 (5) | 83 (5) | 78 (11) |
| 60 | 18 (3) | 82 (14) | 59 (7) | 77(10) | 74 (5) | 84 (5) | 81 (10) |
| 70 | 18 (3) | 82 (14) | 60 (6) | 78 (8) | 75 (5) | 85 (6) | 82 (9) |
| 80 | 18 (2) | 86 (9) | 62 (6) | 81 (8) | 76 (5) | 86 (6) | 84 (8) |
| 90 | 19 (3) | 86 (14) | 62 (6) | 81 (8) | 76 (4) | 86 (5) | 84 (9) |
| 100 | 19 (3) | 86 (14) | 63 (6) | 82 (8) | 77 (4) | 88 (5) | 85 (9) |
| 110 | 19 (3) | 86 (14) | 64 (5) | 83 (7) | 77 (4) | 88 (5) | 86 (9) |
| 120 | 19 (2) | 86 (9) | 64 (5) | 83 (6) | 78 (5) | 89 (6) | 86 (7) |
| 130 | 19 (2) | 86 (9) | 65 (5) | 84 (6) | 78 (4) | 89 (5) | 86 (7) |
| 140 | 19 (3) | 86 (14) | 66 (5) | 86 (6) | 79 (4) | 90 (5) | 87 (8) |
| 150 | 19 (2) | 86 (9) | 66 (4) | 86 (5) | 79 (4) | 90 (4) | 87 (6) |
| 160 | 19 (2) | 86 (9) | 66 (5) | 86 (7) | 79 (4) | 90 (4) | 87 (7) |
| 170 | 20 | 91 (13) | 67 (4) | 87 (6) | 79 (4) | 90 (4) | 89 (8) |
| 180 | 20 | 91 (13) | 67 (4) | 87 (6) | 79 (4) | 90 (4) | 89 (8) |
| 190 | 20 | 91 (9) | 67 (5) | 87 (7) | 80 (3) | 91 (3) | 90 (6) |
| 200 | 20 | 91 (9) | 68 (5) | 88 (7) | 80 (4) | 91 (4) | 90 (7) |
| 210 | 20 | 91 (9) | 68 (4) | 88 (5) | 80 (4) | 91 (4) | 90 (6) |
| 220 | 20 | 91 (9) | 68 (4) | 88 (5) | 80 (3) | 91 (3) | 90 (6) |
| 230 | 20 | 91 (9) | 69 (5) | 90 (6) | 80 (3) | 91 (3) | 91 (6) |
| 240 | 20 | 91 (9) | 69 (4) | 90 (5) | 81 (3) | 92 (3) | 91 (6) |
| 250 | 20 | 91 (9) | 69 (4) | 90 (5) | 81 (3) | 92 (3) | 91 (6) |
| 260 | 20 | 91 (9) | 69 (4) | 90 (5) | 81 (3) | 92 (3) | 91 (6) |
| 270 | 20 | 91 (9) | 70 (4) | 91 (5) | 81 (3) | 92 (3) | 91 (6) |
| 280 | 20 | 91 (9) | 70 (4) | 91 (5) | 81 (4) | 92 (3) | 91 (6) |
| 290 | 20 | 91 (9) | 70 (3.25) | 91 (5) | 81 (3) | 92 (3) | 91 (6) |
| 300 | 20 | 91 (9) | 70 (3) | 91 (4) | 81 (4) | 92 (4) | 91 (6) |
| 310 | 20 | 91 (9) | 70 (4) | 91 (6) | 81 (3) | 92 (3) | 91 (6) |
| 320 | 20 | 91 (9) | 70 (4) | 91 (6) | 81 (3) | 92 (3) | 91 (6) |
| 330 | 20 | 91 (5) | 70 (4) | 91 (6) | 81 (3) | 92 (3) | 91 (5) |
| 340 | 20 | 91 (9) | 71 (3) | 92 (4) | 81 (3) | 92 (3) | 92 (5) |
| 350 | 20 | 91 (9) | 71 (3) | 92 (4) | 82 (3) | 93 (3) | 92 (5) |
| 360 | 20 | 91 (5) | 71 (4) | 92 (5) | 82 (3) | 93 (3) | 92 (4) |
| 370 | 20 | 91 (5) | 71 (4) | 92 (5) | 82 (3) | 93 (3) | 92 (4) |
| 380 | 20 | 91 (9) | 71 (4) | 92 (5) | 82 (3) | 93 (3) | 92 (6) |
| 390 | 20.5 | 93 (5) | 71 (4) | 92 (5) | 82 (3) | 93 (3) | 93 (4) |
| 400 | 20 | 91 (4) | 71 (4) | 92 (5) | 82 (3) | 93 (2) | 92 (4) |
| 410 | 21 | 95 (4) | 71 (4) | 92 (5) | 82 (2) | 93 (2) | 93 (4) |
| 420 | 21 | 95 (4) | 71(3.25) | 92 (4) | 82 (2) | 93 (3) | 93 (4) |
| 430 | 21 | 95 (4) | 71 (4) | 92 (5) | 82 (3) | 93 (3) | 93 (4) |
| 440 | 21 | 95 (4) | 71 (3) | 92 (4) | 82 (3) | 93 (3) | 93 (4) |
| 450 | 21 | 95 (4) | 72 (3) | 94 (4) | 82 (3) | 93 (3) | 94 (4) |
| 460 | 21 | 95 (4) | 72 (3) | 94 (4) | 82 (3) | 93 (3) | 94 (4) |
| 470 | 21 | 95 (4) | 72 (3) | 94 (4) | 82 (3) | 93 (3) | 94 (4) |
| 480 | 21 | 95 (4) | 72 (3) | 94 (4) | 82 (3) | 93 (3) | 94 (4) |
| 490 | 21 | 95 (4) | 72 (2) | 94 (3) | 82 (3) | 93 (3) | 94 (3) |
| 500 | 21 | 95 (4) | 72 (4) | 94 (5) | 82 (3) | 93 (3) | 94 (4) |

**Figure A.1: The mean replicability in all rated items with increasing sample size in the three datasets**


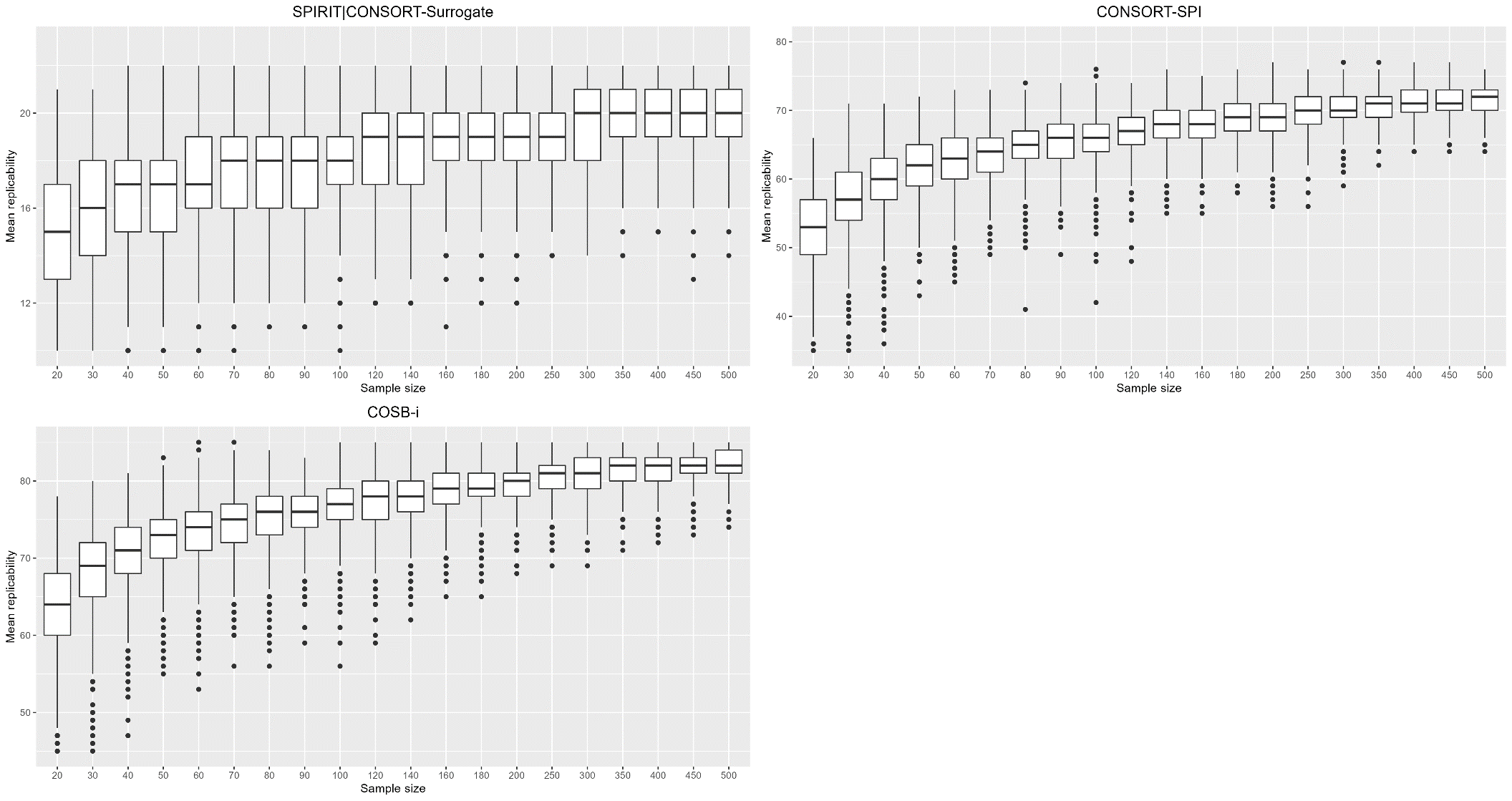


**Supplementary Table A.2: The median (interquartile range) replicability and percentage replicability (variability) in all rated items** **comparing participants from the four types of income level countries in the COSB-i dataset.**

| Sample size | **COSB-i** | | | | | | | |
| --- | --- | --- | --- | --- | --- | --- | --- | --- |
|  | **HIC, N=351** | | **HMIC, N=50** | | **LMIC, N=64** | | **LIC, N=11** | |
|  | Median replicability (IQR) | % Replicability (%variability) | Median replicability (IQR) | % Replicability (%variability) | Median replicability (IQR) | % Replicability (%variability) | Median replicability (IQR) | % Replicability (%variability) |
| 20 | 58 (8) | 66 (9) | 62 (9) | 70 (10) | 57 (9) | 65 (11) | 62 (9) | 70 (10) |
| 30 | 64 (7) | 73 (8) | 65 (8) | 74 (9) | 62 (8) | 70 (9) | 66 (9) | 75 (11) |
| 40 | 67 (6) | 76 (7) | 68 (8) | 77 (9) | 66 (7) | 75 (8) | 68 (7) | 77 (8) |
| 50 | 69 (6) | 78 (7) | 70 (7) | 80 (8) | 68 (7) | 77 (8) | 70 (8) | 80 (9) |
| 60 | 70 (6) | 80 (6) | 71 (6) | 81 (7) | 69 (6) | 78 (7) | 72 (8) | 82 (9) |
| 70 | 72 (5) | 82 (6) | 72 (6) | 82 (7) | 71 (6) | 81 (7) | 73 (7) | 83 (8) |
| 80 | 73 (5) | 83 (5) | 72 (6) | 82 (7) | 72 (6) | 82 (7) | 75 (7) | 85 (8) |
| 90 | 74 (5) | 84 (5) | 73 (6) | 83 (6) | 73 (6) | 83 (6) | 76 (7) | 86 (8) |
| 100 | 74 (5) | 84 (4) | 73 (5) | 83 (5) | 74 (6) | 84 (7) | 76 (7) | 86 (8) |
| 110 | 75 (4) | 85 (6) | 74 (5) | 84 (5) | 75 (5) | 85 (6) | 77 (7) | 88 (8) |
| 120 | 75 (5) | 85 (5) | 75 (5) | 85 (6) | 75 (5) | 85 (6) | 78 (6) | 89 (7) |
| 130 | 76 (4) | 86 (6) | 75 (5) | 85 (6) | 76 (5) | 86 (6) | 79 (7) | 90 (8) |
| 140 | 76 (5) | 86 (5) | 75 (6) | 85 (7) | 76 (4) | 86 (5) | 79 (7) | 90 (8) |
| 150 | 76 (4) | 86 (5) | 75 (5) | 85 (6) | 77 (5) | 88 (6) | 80 (6) | 91 (6) |
| 160 | 76 (3) | 86 (4) | 76 (5) | 86 (6) | 77 (5) | 88 (6) | 80 (7) | 91 (7) |
| 170 | 77 (4) | 88 (5) | 76 (5) | 86 (6) | 77 (5) | 88 (6) | 81 (7) | 92 (7) |
| 180 | 77 (4) | 88 (5) | 76 (5) | 86 (6) | 78 (5) | 89 (6) | 82 (7) | 93 (8) |
| 190 | 77 (4) | 88 (5) | 76 (5) | 86 (6) | 78 (4) | 89 (5) | 82 (7) | 93 (8) |
| 200 | 78 (3) | 89 (4) | 77 (5) | 87 (6) | 78 (4) | 89 (5) | 82 (7) | 93 (8) |
| 210 | 78 (4) | 89 (5) | 77 (5) | 88 (6) | 79 (5) | 90 (6) | 83 (6) | 94 (7) |
| 220 | 78 (4) | 89 (5) | 77 (5) | 88 (6) | 79 (4) | 90 (4) | 83 (6) | 94 (7) |
| 230 | 78 (4) | 89 (5) | 77 (5) | 88 (6) | 79 (4) | 90 (4) | 83 (6) | 94 (7) |
| 240 | 78 (4) | 89 (5) | 77 (4) | 88 (5) | 79 (4) | 90 (4) | 84 (5) | 95 (6) |
| 250 | 78 (3) | 89 (3) | 77 (5) | 88 (6) | 79 (4) | 90 (4) | 84 (5) | 95 (6) |
| 260 | 79 (3) | 90 (3) | 77 (4) | 88 (5) | 80 (4) | 91 (4) | 84 (6) | 95 (7) |
| 270 | 78 (3) | 89 (3) | 78 (5) | 89 (6) | 80 (3) | 91 (3) | 85 (6) | 97 (7) |
| 280 | 79 (3) | 90 (3) | 78 (5) | 89 (6) | 80 (4) | 91 (4) | 85 (5) | 97 (6) |
| 290 | 79 (4) | 90 (4) | 78 (5) | 89 (6) | 80 (4) | 91 (4) | 85 (5) | 97 (6) |
| 300 | 79 (4) | 90 (4) | 78 (5) | 89 (6) | 80 (4) | 91 (4) | 85 (5) | 97 (6) |
| 310 | 79 (4) | 90 (4) | 78 (4) | 89 (5) | 80 (4) | 91 (4) | 86 (4) | 98 (5) |
| 320 | 79 (4) | 90 (4) | 78 (4) | 89 (5) | 80 (4) | 91 (4) | 86 (4) | 98 (5) |
| 330 | 79 (3) | 90 (3) | 78 (4) | 89 (5) | 81 (3) | 92 (3) | 86 (5) | 98 (6) |
| 340 | 80 (4) | 91 (4) | 78 (4) | 89 (6) | 81 (3) | 92 (3) | 86 (4) | 98 (5) |
| 350 | 80 (4) | 91 (4) | 78 (5) | 89 (6) | 81 (3) | 92 (3) | 86 (4) | 98 (5) |
| 360 | 80 (3) | 91 (3) | 79 (5) | 90 (6) | 81 (4) | 92 (4) | 86 (4) | 98 (5) |
| 370 | 80 (4) | 91 (4) | 78 (5) | 89 (6) | 81 (4) | 92 (4) | 86 (4) | 98 (5) |
| 380 | 80 (3) | 91 (3) | 79 (5) | 90 (6) | 81 (4) | 92 (4) | 87 (4) | 99 (5) |
| 390 | 80 (4) | 91 (4) | 79 (4) | 90 (4) | 81 (3) | 92 (3) | 87 (3) | 99 (3) |
| 400 | 80 (4) | 91 (4) | 79 (5) | 89 (6) | 81 (3) | 92 (3) | 87 (3) | 99 (3) |
| 410 | 80 (4) | 91 (4) | 79 (5) | 90 (6) | 81 (3) | 92 (3) | 87 (3) | 99 (3) |
| 420 | 80 (4) | 91 (4) | 79 (4) | 90 (4) | 81 (3) | 92 (3) | 87 (3) | 99 (3) |
| 430 | 80 (4) | 91 (4) | 79 (4) | 90 (4) | 82 (3) | 93 (3) | 87 (3) | 99 (3) |
| 440 | 80 (4) | 91 (4) | 79 (4) | 90 (4) | 82 (3) | 93 (3) | 87 (3) | 99 (3) |
| 450 | 80 (4) | 91 (4) | 79 (4) | 90 (4) | 82 (3) | 93 (3) | 87 (3) | 99 (3) |
| 460 | 80 (3) | 91 (3) | 79 (4) | 90 (4) | 82 (3) | 93 (3) | 87 (2) | 99 (2) |
| 470 | 80 (3) | 91 (3) | 79 (4) | 90 (4) | 82 (3) | 93 (3) | 87 (2) | 99 (2) |
| 480 | 80 (4) | 91 (4) | 79 (4) | 90 (4) | 82 (3) | 93 (3) | 87 (2) | 99 (2) |
| 490 | 81 (3) | 92 (3) | 79 (4) | 90 (4) | 82 (4) | 93 (3) | 88 (2) | 100 (2) |
| 500 | 81 (3) | 92 (3) | 79 (4) | 90 (4) | 82 (3) | 93 (3) | 88 (2) | 100 (2) |

**Figure A.2: The median number of items reaching consensus with increasing sample size in the three datasets**
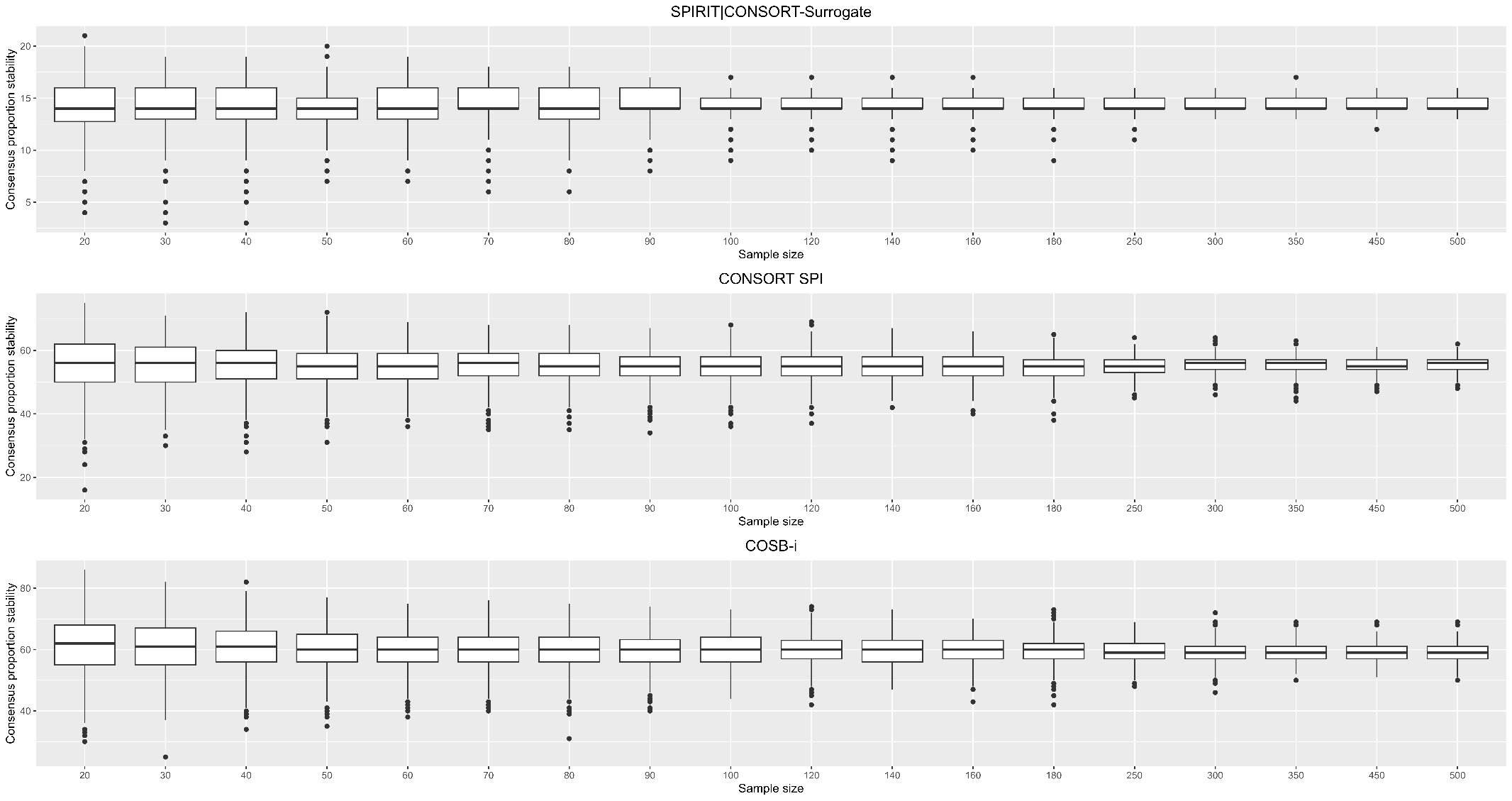

Supplement: Supplementary data [file EMS207315-supplement-Supplementary_data.docx]
